# Molecular Basis and Natural History of Medullary Thyroid Cancer: It is (Almost) All in the *RET*

**DOI:** 10.3390/cancers15194865

**Published:** 2023-10-05

**Authors:** Nicolas Sahakian, Frédéric Castinetti, Pauline Romanet

**Affiliations:** 1 Aix Marseille Univ, APHM, INSERM, MMG, La Conception University Hospital, Department of Endocrinology, Marseille, France; nicolas.sahakian@ap-hm.fr (N.S.); frederic.castinetti@ap-hm.fr (F.C.); 2 Aix Marseille Univ, APHM, INSERM, MMG, La Conception University Hospital, Laboratory of Molecular Biology, Marseille, France

**Keywords:** medullary thyroid carcinoma, multiple endocrine neoplasia type 2, *RET*, germline or somatic genetic mutation, *RET* tyrosine kinase inhibitor, predictive biomarker

## Abstract

**Simple Summary:**

Medullary thyroid carcinoma (MTC) is a rare neoplasm supported by a strong genetic determinism. This review summarizes the genetic landscape of MTC at both germline and somatic levels to understand the molecular basis and the natural history of the tumour, mainly but not exclusively, linked to *RET* proto-oncogene genetic abnormalities. *RET* is a tyrosine kinase receptor that represents a therapeutic target with encouraging results. However, some *RET* genetic variations could lead to treatment resistance.

**Abstract:**

Medullary thyroid cancer (MTC) is a rare disease, which can be either sporadic (roughly 75% of cases) or genetically determined (multiple endocrine neoplasia type 2, due to REarranged during Transfection *RET* germline mutations, 25% of cases). Interestingly, *RET* pathogenic variants (mainly M918T) have also been reported in aggressive forms of sporadic MTC, suggesting the importance of *RET* signalling pathways in the pathogenesis of MTC. The initial theory of *RET* codon-related MTC aggressiveness has been recently questioned by studies suggesting that this would only define the age at disease onset rather than the aggressiveness of MTC. Other factors might however impact the natural history of the disease, such as *RET* polymorphisms, epigenetic factors, environmental factors, *MET* (mesenchymal–epithelial transition) alterations, or even other genetic alterations such as *RAS* family (*HRAS*, *KRAS*, *NRAS*) genetic alterations. This review will detail the molecular bases of MTC, focusing on *RET* pathways, and the potential mechanisms that explain the phenotypic intra- and interfamilial heterogeneity.

## 1. Introduction

Thyroid carcinoma is the most frequently occurring endocrine cancer, with a reported worldwide prevalence of about 1.9 million cases and an estimated age-standardized incidence rate of 6.6/100,000 [1]. Different from the most common papillary thyroid carcinoma (PTC), which accounts for over 90% cases, medullary thyroid carcinoma (MTC) is a rare neoplasm originating from the C-cells of the thyroid first described by Hazard et al. in the late 1960s [2]. With an incidence of 0.19/100,000 per year and a prevalence of 3.8/100,000 [3], MTC represents up to 5% of all thyroid gland carcinomas and shows a strong genetic determinism, at both the germline and somatic levels. While roughly 75% cases are sporadic, 25% are hereditary and are a part of multiple endocrine neoplasia type 2 (MEN2), which is caused by REarranged during Transfection (*RET*) germline mutations [4,5]. MTCs are encountered in nearly 100% of MEN2 cases. *RET* is a tyrosine kinase receptor [6] and plays a central role in MTC development, not only in hereditary MTCs but also in sporadic cases, in which somatic *RET* pathogenic variants have also been reported in aggressive forms and been shown to correlate with worse outcomes, suggesting the importance of *RET* signalling pathways in the pathogenesis of MTC [6]. *RET* is greatly involved in all aspects of MTC management: (1) discovery of a *RET* pathogenic germline variant leads to the diagnosis of MEN2, (2) presence of a *RET* pathogenic variant, at both the germline and somatic levels, predicts tumour aggressiveness and, lastly, (3) presence of a *RET* pathogenic variant enables specific therapy such as tyrosine kinase inhibitor to be employed. Other factors can however impact the natural history of the disease, including *RET* polymorphisms, epigenetic factors, environmental factors, or even other genetic alterations, such as *MET* (mesenchymal–epithelial transition), *RAS* family (*HRAS*, *KRAS*, *NRAS*), or *CDKN* family (cyclin dependant kinase inhibitor) genetic alterations. This review will detail the molecular bases of MTC, focusing on the *RET* pathways and potential mechanisms that explain phenotypic heterogeneity.

## 2. Methodology

The authors identified articles published in English up to June 2023, using the U.S. National Library of Medicine (PubMed^®^; https://www.ncbi.nlm.nih.gov/pubmed (accessed on 30 June 2023) database, performing a systematic search using the following terms: “multiple endocrine neoplasia type 2”, “MEN2”, “medullary thyroid carcinoma”, “MTC”, “hereditary”, “familial”, “sporadic”, “REarranged during Transfection”, “*RET*”, “structure”, “function”, “mutation”, “variant”, “polymorphism”, “germline”, “somatic”, “RAS”, “HRAS”, “KRAS”, “NRAS”, “MET”, “CDKN”, “epigenetic”, “outcome”, “prognosis”, “mortality”, “metastasis”, “cabozantinib”, “vandetanib”, “selpercatinib”, “pralsetinib”, “zeletinib”, “tyrosine kinase inhibitor”, “TKI”, “*RET* specific TKI”, “resistance”, and “TKI resistance”. A secondary review of the reference lists and subsequent manuscripts citing previously published papers led to the identification of additional relevant articles.

## 3. Molecular Basis of the *RET* Pathway

- *RET* proto-oncogene and *RET* protein structure

**Figure 1:**
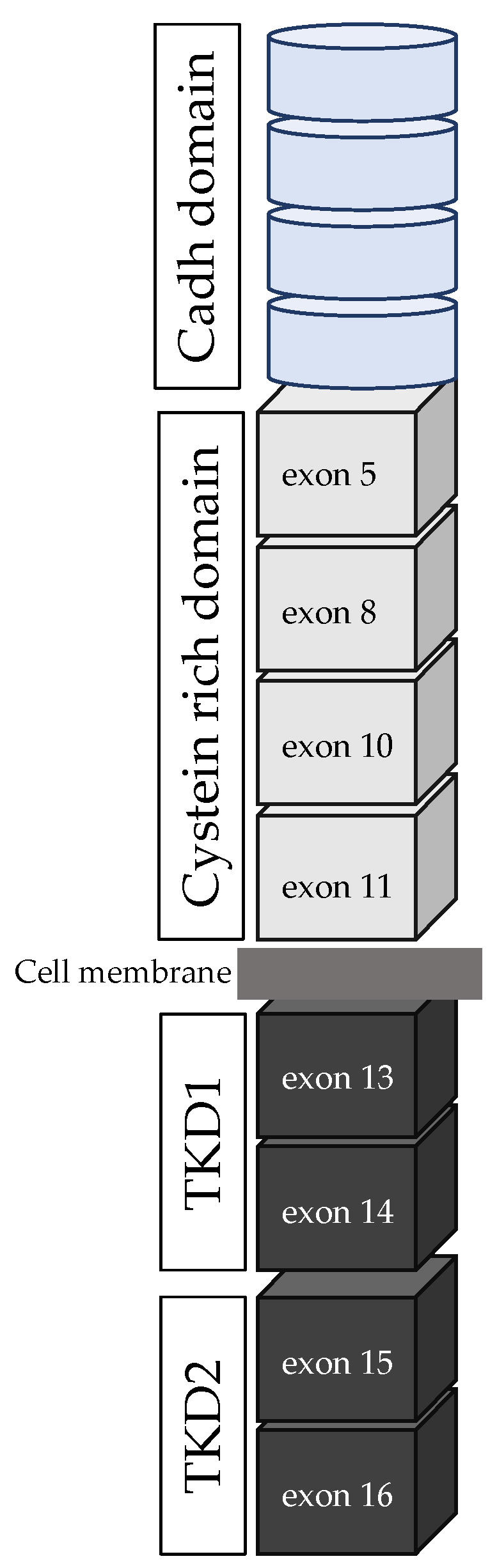
RET structure. Soft blue cylinder represents the cadherin domain, and soft grey boxes represent the cysteine-rich domain, including exons 5, 8, 10, and 11. The two grey lines represent the cell membrane. Grey boxes represent the intracellular part, with both tyrosine kinase domain 1 (exons 13 and 14) and tyrosine kinase domain 2 (exons 15 and 16). Cadh domain: cadherin domain; CRD: cysteine-rich domain; TKD: tyrosine kinase domain.

The REarranged during Transfection (*RET*) proto-oncogene was identified in 1985 as a novel transforming gene in NIH3T3 cells upon transfection with DNA isolated from human lymphoma cells [7]. *RET* is located on chromosome 10q11.2 and is composed of 20 exons (48 Kb). It encodes a transmembrane protein belonging to the cadherin superfamily and acts as a tyrosine kinase (TK) receptor [8]. As a membrane receptor, *RET* has three domains: an extracellular domain, a transmembrane domain, and an intracellular domain, which contains the tyrosine kinase domain (Figure 1) [7,9,10]. The extracellular part consists of a region homologous to the cadherin family of cell adhesion molecules (four cadherin-like domains) and a large cysteine-rich domain (with 16 cysteine residues in a stretch of 120 amino acids). The intracellular domain is divided into two tyrosine kinase subdomains separated by 28 amino acids (TK1 and TK2). These subdomains contain the tyrosine residues that are phosphorylated during receptor activation and are involved in the activation of downstream intracellular pathways. *RET* is subject to alternative splicing in the 3′ region, producing three different protein isoforms that contain 9 (*RET*9), 43 (*RET*43), and 51 (*RET*51) additional amino acids in the C-terminal tail, downstream from glycine 1063 (*RET*9: 1072 amino acids, *RET*43: 1106 amino acids, and *RET*51: 1114 amino acids) [9]. Both *RET*9 and *RET*51 are the major forms found in vivo and have molecular weights between 150 and 170 KDa, depending on the degree of N-glycosylation [10].

b. *RET* canonical signalling pathway

**Figure 2:**
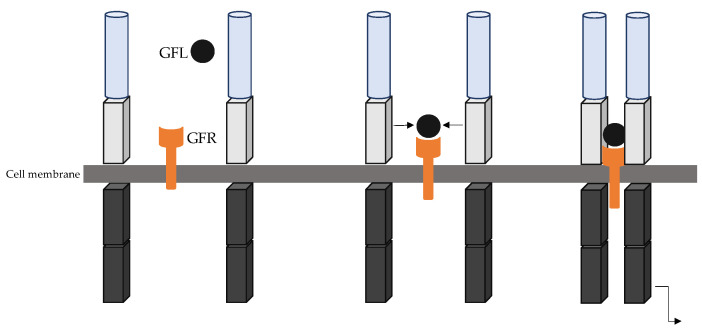
RET canonical pathway. Soft blue cylinder represents the cadherin domain and soft grey boxes represent the cysteine-rich domain. The two grey lines represent the cell membrane. Grey boxes represent the tyrosine kinase domain. GLF: glial ligand; GFR: glial receptor.

*RET* is mainly expressed in cells derived from the neural crest, including thyroid C-cells, chromaffin cells of the adrenal medulla, and the enteric autonomic plexus. *RET* is essential for various biological functions including enteric nervous system development, kidney and lower urinary tract development, as well as spermatogenesis [11]. The *RET* receptor is activated through a complex formed by the glial cell line-derived neurotrophic factor (GDNF) family of ligand (GFL) (GDNF family, such as Neurturin, Artemin, and Persephin) and co-receptors. The GFR co-receptors are classically bound to the plasma membranes within lipid rich rafts, but also occur in a soluble form and both can then activate *RET* in two distinct forms of activation. Under normal conditions, *RET* ligand-dependent activation is mediated by Ca^2+^ ions binding to cadherin-like domains and the interaction of GFR co-receptors and their respective GFL ligand. The GFR ligand complex, together with the extracellular domain of *RET* leads to *RET* dimerization and autophosphorylation of the intracellular tyrosine residue (Figure 2) [12,13]. Once activated, *RET* initiates different intracellular downstream pathways involved in the regulation of crucial processes such as differentiation, survival, proliferation, and migration [11].

c. *RET* oncogene

**Figure 3:**
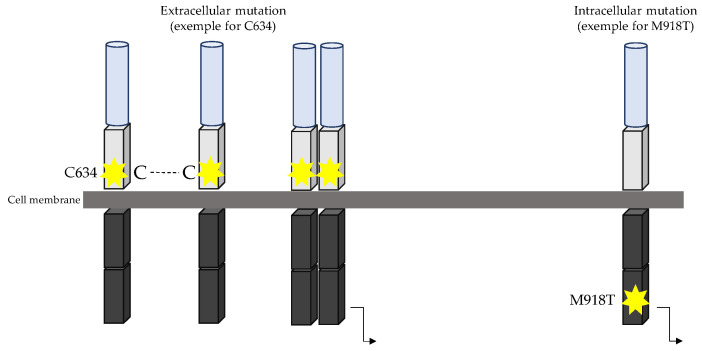
RET oncogenic activation in both extracellular and intracellular *RET* mutant responsible for MEN2. Soft blue cylinder represents the cadherin domain and soft grey boxes represent the cysteine-rich domain. The two grey lines represent the cell membrane. Grey boxes represent the tyrosine kinase domain. Extracellular mutation occurring in cysteine-rich domain leads to aberrant intermolecular disulphide bond, leading to *RET* dimerization and activation without ligand. Intracellular mutation occurring in tyrosine kinase domain leads to *RET* autophosphorylation, particularly in *RET* M918T in which *RET* is activated as a monomer. Yellow stars represent the *RET* mutation localisation.

In MTC, several activating mutations can confer oncogenic properties to *RET*, located in both intra- and extra-cellular domains. The molecular mechanism by which *RET* activating mutations drive the neoplastic process was determined by Santoro and co-workers [14]. Under normal conditions, *RET* is only activated in the presence of GFR/GFL complex, which on binding to the *RET* receptor drives its dimerization and autophosphorylation of the intracellular signalling pathway. In vivo studies have highlighted that *RET* mutations C634R and M918T lead to constitutive phosphorylation of tyrosine, and their in vitro kinase activity was significantly higher than that of the wild-type protein [15]. Most mutations that occur in the extracellular domain are located in the cysteine-rich domain on exon 10 and exon 11. This domain contains cysteine residues, leading to formation of disulphide bonds necessary for the correct conformation of *RET* receptors. Mutations occurring in codons 609, 611, 618, 620, 630, and 634, which all code for a cysteine residue, lead to the misconformation of *RET* and allow self-dimerization without a ligand. Mutations affecting one of six cysteines located in the juxtamembrane domain of the *RET* receptor led to the replacement of the Cys residue with an alternate amino acid. Functional studies have shown that each of these mutations induced constitutive catalytic activity due to aberrant disulphide homodimerization of *RET* [16,17]. *RET* proteins with Cys 634 mutation were constitutively phosphorylated on tyrosine and their in vitro kinase activity was significantly higher than that of the WT protein [15]. Mutations in exon 11 (codon 630 and 634) lead to higher *RET* activation compared to mutations within exon 10 (codons 609, 611, 618, and 620) [16]. While extracellular mutations still required *RET* dimerization for signal transduction, intracellular mutations within exon 16 (*RET* M918T) did not [15,18,19] (Figure 3). Moreover, studies have highlighted that M918T is phosphorylated early and interacts with partners inducing downstream signalling before reaching the cell membrane [20].

## 4. Genetic Drivers of Hereditary and Sporadic MTC

- Hereditary MTC

**Figure 4:**
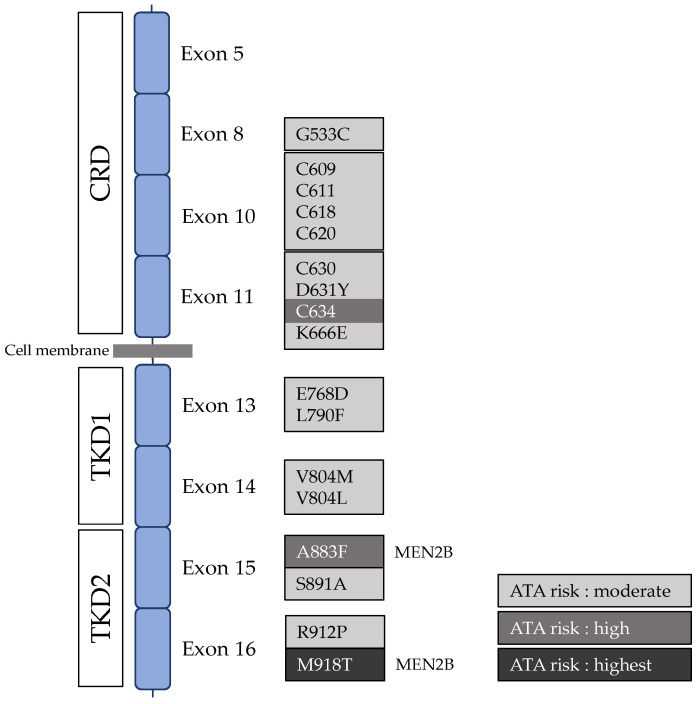
Representation of the most common reported *RET* mutations and their localization. CRD: cysteine-rich domain; TKD1: tyrosine kinase domain 1; TKD2: tyrosine kinase domain 2.

The concurrence of thyroid carcinoma and pheochromocytoma was first reported in 1961 by Sipple [21]. Later in 1968, Steiner et al. reported a family with an association of thyroid carcinoma, pheochromocytoma, hyperparathyroidism, and Cushing’s syndrome [22]. To describe this syndrome, and mirroring the term previously used by Wermer to characterise multiple adenomatosis of endocrine glands [23], the term multiple endocrine neoplasia type 2 (MEN2) was first coined. As previously described, the *RET* gene was identified as responsible for MEN2 in the late 1990s [24]. At first, a genotype–phenotype correlation was suggested [22]. The majority of MEN2A (OMIM #171400) results from missense mutations within the extracellular domain, particularly in the cysteine-rich domain and mainly involving codon 634 [4,24,25,26], whereas MEN2B (OMIM #162300) is almost exclusively caused by a specific exon 16 mutation (c.2753T > C, p.Met918Thr) [4,25,26,27]. Apart from these most commonly reported mutations, several other variants both in the extracellular [28,29,30,31,32,33] and intracellular domains [34,35,36,37,38] have been reported as being responsible for MEN2 (*RET* variants and exon localisation are summarized in Figure 4).

At the germline level, rare indel mutations can also be found to be responsible for MEN2-associated disease. Pathogenic mutations in the *RET* proto-oncogene have been successively reported since 2009 in an open access database [39] and are now available from the online ARUP MEN2 database [40].

MTC occurs in roughly 100% of MEN2 cases and may be more or less aggressive, depending on the *RET* mutation that leads to MEN2. *RET* mutations are thus classified into three risk grades (moderate, high, and highest risk), according to the risk of aggressive MTC [41]. This classification is currently the basis for international guidelines for MEN2-related MTC management proposed by the American Thyroid Association (ATA) [4]. Though the genotype–phenotype correlation is well-accepted, the theory of *RET* codon-related MTC aggressiveness has recently been questioned. In a large monocentric observational study involving 262 patients with moderate-risk (*n* = 127) and high-risk (*n* = 135) germline *RET* mutations, Voss et al. reported that both moderate- and high-risk patients had similar overall survival (OS), metastatic disease after MTC diagnosis, and clinical outcomes, suggesting that moderate- and high-risk *RET* mutations do not predict MTC aggressiveness [42]. In this study, lymph node invasion was frequently described in patients with moderate- and high-risk *RET* mutations (53/127, 42%, and 48/135, 36%, respectively), but not distant metastatic disease (10/127, 8%, and 7/135, 5%, respectively). Finally, this study demonstrates that metastatic disease is not more frequent in MEN2 patients with high-risk *RET* mutations, i.e., mutations in codon C634, than in MEN2 patients with moderate-risk *RET* variants. Thus, the authors suggested that *RET* mutation would only define the age at disease onset rather than the aggressiveness of MTC [42]. However, this study was limited by differences in the duration of follow-up between the two groups, as patients with a moderate-risk *RET* variant had a shorter follow-up compared to patients with a high-risk variant (median follow-up 6.5 vs. 11.5 years, respectively). Moreover, age at last follow-up was lower in patients with high-risk variants compared to patients with moderate risk (34.5 vs. 48.5 years, respectively) [43]. Two other independent studies have published similar findings. In a cohort comparing 122 moderate-risk and 120 high-risk MTC, Raue et al. reported that both had the same disease stage at the time of diagnosis (in particular, five patients in both groups (4%) exhibited stage IV disease at onset). At the last follow-up, both moderate-risk and high-risk groups showed the same remission rate (56% vs. 61%), the same rate of metastatic disease (14% vs. 13%), and the same disease-specific death (7% vs. 7%). Interestingly, while high-risk patients were younger at the time of diagnosis of stage III disease compared to moderate-risk patients (30.4 ± 13.0 years vs. 41.7 ± 15.3 years), the mean interval from stage I to III did not differ (12.7 years vs. 10.1 years). These results support the previously proposed hypothesis that MEN2A *RET* pathogenic variants can determine the age of onset of MTC, but that MTC is an equally aggressive tumour in both moderate- and high-risk groups after its initial onset. However, the age at surgery and the duration of follow-up differed between moderate- and high-risk groups, as being younger (23.0 ± 15.7 years vs. 35.3 years) and longer (15.8 ± 9.5 years vs. 10.8 ± 9.0 years), respectively. Moreover, successive therapeutic lines employed in the patients were not available and possibly varied between the two groups [44]. Machens et al. investigated the progression from node-negative to node-positive MTC according to the *RET* risk category. Considering 387 patients, 201 with node-negative and 186 with node-positive MTC, the author showed that tumour progression to node metastasis was similar in both groups, taking 8.6 to 9.1 years in the moderate-risk group and 13.6 years in the high-risk MTC patients [45]. However, this study compared the age at thyroidectomy between the groups and not the time between thyroidectomy and node progression in the same patient. Other authors have reported atypical cases of late onset MEN2-related tumours despite carrying the high-risk *RET* variant C634 [46,47], highlighting that other factors might modify the natural history of MEN2-related MTC.

b. Sporadic MTC

Sporadic MTC is defined as MTC in patients without the germline *RET* mutation, other MEN2-related manifestations, such as pheochromocytoma and hyperparathyroidism, or familial history suggestive of MEN2.

- Somatic *RET* mutations are a major factor in sporadic MTC tumourigenesis.

In the late 1990s, Marsh et al. showed that two thirds of sporadic MTCs exhibited a somatic mutation at codon 918 (M918T) [48]. These data were recently confirmed by NGS-targeted sequencing in a large cohort of 148 sporadic MTCs. Apart from codon 918 (*RET* M918T) [6,48,49], somatic events may occur in codon 634 [49,50] and other MEN2-related variants [48,51,52]. Other kinds of mutation, such as indels involving critical cysteine residues, may lead to *RET* oncogenic activation that is responsible for MTC (Table 1). Somatic *RET* mutations, particularly *RET* M918T, are related to the MTC phenotype. *RET* M918T mutated MTCs present a more advanced stage at diagnosis and have worse outcomes [6,49,53]. The presence of *RET* mutations, both together and when considering M918T alone, correlates to an advanced stage of the disease (*p* = 0.0025), a higher tumour (T) category (*p* < 0.0001), and the presence of both lymph-node (N) (*p* = 0.0021) and distant metastases (M) (*p* = 0.0073) [54]. In a large meta-analysis including 964 sporadic MTCs from 23 studies, the sporadic MTCs with somatic *RET* mutation were associated with a younger age at diagnosis, a higher proportion of pT3/T4 (OR = 2.31, 95%CI [1.55–3.45], I^2^ = 0%), with an increased risk of lymph node metastasis (OR = 3.61, 95%CI [2.33–5.60, I^2^ = 0%) and with a higher propensity for distant metastasis (OR = 2.85, 95%CI [1.64–4.94], I^2^ = 0%) compared to MTCs with wild-type *RET* [55]. Finally, in sporadic MTCs, *RET* mutation was found to be an indicator of poor prognosis, with an increased risk of both tumour recurrence (OR = 3.01, 95%CI [1.65–5.48], I^2^ = 41%) and patient death (OR = 2.43, 95%CI [1.06–5.57], I^2^ = 33%) [55]. Romei et al. reported that the majority of sporadic MTCs that were advanced and metastatic carried a *RET* mutation, mainly M918T (93.8%) [56]. The prevalence of somatic *RET* M918T mutation frequency increases with tumour size, from 11.3% in microMTCs to 58.8% in MTCs larger than 30 mm [57]. Even though the role of missense mutations is well-known, the role of other molecular alterations remains uncertain. A recent study revealed that *RET* indels are not uncommon, occurring in up to 17.4% of MTCs, and that these correlate with aggressive disease [58].

*RET* copy number alterations (CNAs) are found in 13 to 30% of hereditary and sporadic MTCs [66,67]. The presence of *RET* mutation at both the germline or somatic level was more frequent in tumours with *RET* CNAs compared to those without (83.3% vs. 42.5%, *p* = 0.003) [68]. Another study showed that *RET* amplifications only affect tumours carrying mutations, not playing a role in tumourigenesis but potentiating the transforming activity of the *RET* oncogene [69]. Furthermore, the prevalence of *RET* CNAs was significantly higher in advanced stages of the disease compared with lower stages (66.7% vs. 33.3%, *p* = 0.003) (*p* = 0.003) and was associated with poorer outcomes (*p* = 0.005) [68].

Taken together, these data highlight that somatic *RET* mutations appear to be the main oncogenic driver in sporadic MTCs, with *RET* M918T being the most common variant and being correlated with MTC aggressiveness. However, in addition to *RET* somatic mutations and CNAs, other oncogenes commonly involved in cancer pathogenesis have also been investigated in sporadic MTCs.

- *RAS* oncogene in sporadic MTC

Several studies have shown that the human *RAS* gene family plays a crucial role in the development of MTCs. The *RAS* oncogene is the second main oncogenic driver after *RET* in sporadic MTCs. The three *RAS* oncogenes encode three 21 KDa monomeric distinct proteins (H-RAS, N-RAS, K-RAS), which act as GTPases that control key signal transduction cascades including the Raf/MEK/ERK and PI3Kinase/Akt pathways [70,71]. Activating mutations in specific hotspots on the *RAS* genes are found in about 30% of all human cancers. In thyroid neoplasia, *RAS* gene point mutations, mainly in *NRAS*, are detected in benign and malignant tumours that arise from the follicular epithelium. Through a large exome study, Agrawal et al. performed tumoural whole exome sequencing on a total of 57 MTCs (36 sporadic) and reported that overall 91% of all tumours had *RET* (75%), *HRAS* (12%), or *KRAS* (4%) mutations [72]. While somatic *RAS* mutations were found in 10 to 70% of *RET-*negative sporadic MTCs, the association of both *RET* and *RAS* somatic mutations is rare [73,74,75]. Moreover, no *RAS* mutations were found in hereditary MTCs [76], suggesting that activation of *RAS* and *RET* genes are mutually exclusive and represent alternative genetic events in sporadic MTC tumourigenesis. Most *RAS* mutations correspond to mutation hot spots in exons 2 and 3, but a smaller proportion of them were detected in exon 4. Compared to WT *RAS* sporadic MTCs, patients with *RAS*-mutant MTCs showed no significant differences between the age at diagnosis, tumour size, lymph node invasion, and distant metastasis. Though rarely reported in studies, the presence of *RAS* mutations in MTCs was not an indicator for tumour relapse (OR = 0.57, 95%IC [0.23–1.42], I^2^ = 0%) [55]. Thus, patients harbouring a sporadic MTC carrying a *RAS* mutation appear to have a better prognosis than patients harbouring a *RET* mutation [54]. While *RET* and *RAS* oncogenes are present in about 50% to 90% of sporadic MTCs, most of the remaining cases appear to still lack a genetic driver [54,77].

- *CDKN*s and tumour suppressor genes in sporadic MTCs

The members of the CDKN2 family (CDKN2A [p15], CDKN2B [p16], CDKN2C [p18], and CDKN2D [p19]) are cyclin dependant kinase inhibitors that block cell cycle progression by interacting with CDK4 or CDK6 to prevent activation of the Cyclin D–CDK4/6 complex. The loss of CDKNs induces increased and uncontrolled phosphorylation of retinoblastoma protein (RB) and unregulated progression through the S phase of the cell cycle. The loss of chromosome regions 1p32 and 19p13, encoding *CDKNs*, are relatively frequently found in sporadic MTCs (39% and 24%, respectively). Thus, several studies have investigated the putative role of the CDKN/RB pathway in MTC. In a study including 62 sporadic MTCs, *CDKN2C* loss was observed in 19% (12/62) of cases, including six MTCs with a somatic *RET* M918T mutation. *CDKN2C* loss was associated with a higher stage MTC at diagnosis (*p* = 0.0009) and a worse outcome compared to both MTC with or without somatic *RET* M918T mutation and exhibited a lower median overall and disease specific survival. Interestingly, overall survival was lower in patients with a combination of both somatic *RET* M918T and somatic *CDKN2C* copy loss, suggesting a synergistic effect [78]. In a recent study involving 58 sporadic MTCs (40 with somatic *RET* mutation and 10 with somatic *RAS* mutation), *CDKN2C* loss correlated with shorter time to distant metastatic disease (1.59 years vs. 5.18 years, *p* = 0.03) while *RET* mutations did not reach significance (2.83 years vs. 6.60 years, *p* = 0.5) [79]. In vivo preclinical models have assessed the role of loss of *CDKN* in *RET*-mutant MTC. MEN2B (*RET* M918T) mice with *Cdkn2c* knock-out (*Cdkn2c*^−/−^) developed MTC at a greatly increased incidence compared to MEN2B (*RET* M918T) *Cdkn2c*^+/−^ mice and with a higher proliferation rate, suggesting that *Cdkn2c* loss increases the risk of MTC and enhances MTC progression, in synergy with the *RET* oncogene [80]. Aside from *CDKN2C* copy loss, Van Veelen and colleagues identified somatic mutations in *CDKN2C* in four MTCs. In vitro analysis showed that the *CKDN2C* mutant is less stable than the wild type and its interaction with CDK4/6 is impaired, leading to reduced inhibition of cell growth [81].

Polymorphisms in *CDKN1B* and *CDKN2A* were found to be over- and under-represented, respectively, in 45 sporadic MTCs compared to 98 controls (62.2% vs. 40.2%, *p* = 0.038 and 15.6% vs. 32.6%, *p* = 0.0075, respectively) [82]. In this study, *CDKN1B* and *CDKN2A* polymorphisms were found to contribute 11% of the total risk of developing a sporadic MTC and, when combined with *CDKN2C* polymorphisms, to be associated with aggressiveness. Interestingly in this study, only patients with *CDKN2B* polymorphisms exhibited metastatic disease (36.3% vs. 0%, *p* = 0.0261) [82]. In non-hereditary tumours, WT *CDKN2C* displays a higher rate of lymph node metastasis compared to the homozygous variant genotype (83.8% vs. 40%, *p* = 0.03) [83]. *CDKN1B*^WT^/*RET^mut^* MTCs also show more aggressive behaviour, while the *CDKN1B* V109G polymorphism is associated with better outcomes after initial surgery compared to the WT allele [84].

Finally, a protective effect of *CDKN1A* SNP (p.Ser31Arg) was found in both hereditary (n = 77) and non-hereditary MTCs (n = 361) (OR = 0.53, 95%CI [0.36–0.78], *p* = 0.001) [85].

- Other genetic abnormalities in sporadic MTC

c-MET (mesenchymal–epithelial transition factor) and its ligand, hepatocyte growth factor (HGF), belong to the MET family and act as a receptor tyrosine kinase that is expressed on the membrane of various epithelial cells. The HGF/c-MET axis, which can interact and cooperate with other types of tyrosine kinases, can stimulate various downstream signalling pathways in tumour cells, such as PI3K/AKT, JAK/STAT, Ras/MAPK, SRC, and Wnt/β-catenin [86]. The HGF/c-MET pathway is activated in most papillary thyroid cancers [87,88,89,90,91] and is correlated with PTC aggressiveness [91,92]. Activation of *MET* mutations, at both the germline and somatic levels, and gene amplification have been reported in several cancers [93,94], including thyroid neoplasms (in which the main mutation is *MET* T1010I in exon 14) [95]. Two studies have reported that *MET* T1010I variant of uncertain significance was identified in about 3 to 5% of sporadic MTCs [54,95]. Ciampi et al., in a study involving 148 sporadic MTCs, reported that five patients had a *MET* variant and two patients had no other identified driver of tumourigenesis [54]. Nevertheless, HGF and c-MET are expressed in most MTC cases, generally co-expressed [96], but do not show a clear correlation with clinical or pathological features [96]. In view of these data, *MET* mutation does not seem to play a crucial role in MTC tumourigenesis though *MET* gene amplification may act as an additional driver.

Activating transcription factor 4 *(ATF4)* is a crucial negative regulator of *RET* tyrosine kinase receptor in MTC. In mice, *Atf4*-targeted deletion has been shown to result in C-cell hyperplasia (CCH), *Atf4* over-expression, and decreased MTC cell survival, and it inhibited the activation of the *RET* downstream pathway [97]. *RET* negatively regulates ATF4-mediated apoptosis [98]. ATF4 expression is decreased or lost in about half of human MTCs, compared with normal thyroid follicular cells [97] and inversely correlates with an increase in MTC stage [99]. Low ATF4 expression is associated with poor MTC outcomes [97] and has a significant negative impact on median survival when compared to high protein expression (*p* < 0.001). The combination of somatic *RET^M918T^* and low ATF4 protein levels further decreased overall survival [97,100] and was associated with increased 5-year recurrence, while higher ATF4 levels were significantly correlated to better 5-year survival [99].

The *BRAF* gene belongs to the RAF family of protein kinase that are key components of the MAPK signalling pathway, mediating cell growth, differentiation, and survival. While *BRAF* (mainly *BRAF* V600E) is a well-known oncogenic variant involved in various cancers, such as melanoma [101] and papillary thyroid cancer [102], in sporadic MTCs, *BRAF* V600E has rarely been reported [73,76,77,103,104,105,106] and appears to be a distant marginal driver.

In an independent series of 51 MTCs, Araujo et al. reported focal recurrent copy number gains involving the DLK1 (delta like non-canonical Notch ligand 1) and AIFM3 (apoptosis inducing factor mitochondria associated 3) genes. The functional relevance of CNA was also assessed by in silico analysis, and CNA status was found to be correlated with protein expression (DLK1, *p* = 0.01), tumour size (DLK1, *p* = 0.04), and tumour staging (AIFM3, *p* = 0.01 and DLK1, *p* = 0.05). These data provide a novel insight into MTC biology and suggest a common CNA landscape, regardless of whether the tumour is a sporadic or hereditary MTC [107].

Other mutations, such as *AKT1* or *CTNNB1*, are unlikely to be mutation drivers in sporadic MTC [106].

**Figure 5:**
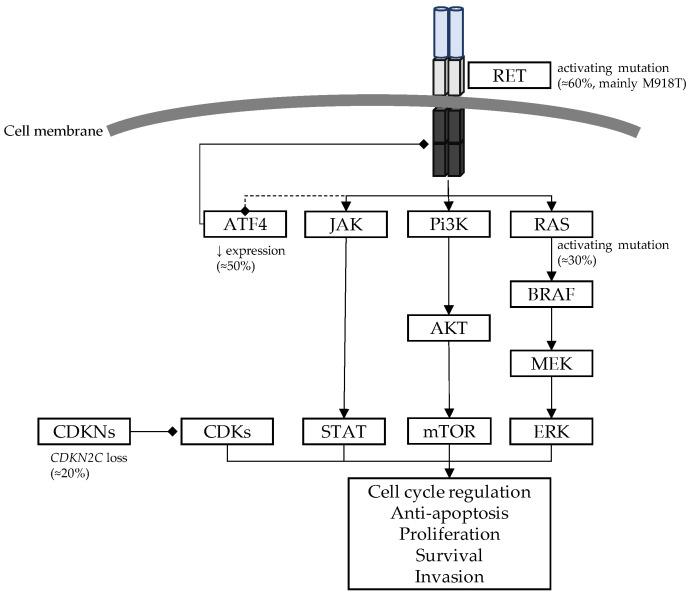
Main oncogenic drivers in sporadic MTC with percentages of most commonly involved genes. MEK: mitogen-activated protein kinase; ERK: extracellular signal-regulated kinases; Pi3K: phosphoinositide 3-kinases; AKT: protein kinase B; mTOR: mammalian target of rapamycin; ATF4: activating transcription factor 4; CDKs: cyclin dependent kinases; CDKNs: cyclin dependent kinase inhibitors.

The main oncogenic drivers involved in sporadic MTCs are summarized in Figure 5.

## 5. Modifiers of the Natural History of MTC

- *RET* single nucleotide polymorphisms and MTC

In recent years, several *RET* single nucleotide polymorphisms have been suspected to be involved in *RET*-mutated MTCs, principally the G691S (exon11, c.2071G>A, p.Gly691Ser, rs1799939), L769L (exon 13, c.2307G>T, p.Leu769=, rs1800861), S836S (exon 14, c.2508C>T, pSer836=, rs1800862), and S904S (exon 15, 2712C>G, p.Ser904=, rs180086) alleles (allelic frequencies from gnomADv2.1 are listed in Table 2). The G691S and S904S *RET* polymorphisms are in linkage disequilibrium and co-segregate, so these results have been grouped together and referred to as the *RET* G691S/S904S haplotype [108,109,110].

Their effect on the development and progression of MTC is not totally convincing, and the explanation of their mechanism remains speculative. For example, *RET* mRNA levels in MTC specimens showed no difference between patients with and without *RET* G691S/S904S variants [108]. In silico analysis revealed that the *RET* G691S variant did not modify *RET* mRNA [111] but appears to introduce two new phosphorylation targets at Serine 686 and 691 and abolishes a phosphorylation site at Tyrosine 687, increasing activation of the RAS/ERK pathway [112]. Functional studies of the *RET* G691S variant showed that it does not exhibit transforming activity per se, but appeared to enhance the oncogenic properties of pathogenic *RET* mutations [113,114,115]. In transfection studies, the *RET* double mutant (G691S/S891A or G691S/K666E) activated the ERK1/2 pathway more than a single pathogenic mutant [114,115]. Other authors have suggested that the *RET* G691S polymorphism could have a cooperative action on *RET* dimerization in MEN2A, with the pathogenic *RET* mutation affecting the extracellular cysteine-rich domain and particularly codon 634 [114]. The clinical relevance of *RET* polymorphisms has been examined in several studies with inconsistent results.

- *RET* G691S/S904S (rs1799939/rs1800863)

*RET* G691S polymorphism was shown to have a modifier effect on the age at onset of MTC in MEN2A families [114,115,116]. In a large Indian study, Mishra et al. identified that the *RET* S904S synonymous variant was over-represented in hereditary MTCs (53.2%) when compared to controls (41.7%) (OR = 2.82, 95%CI [1.64–4.86], *p* < 0.001) [85].

In sporadic MTCs, *RET* G691S polymorphism allele frequency was found to be higher compared to controls (27.83% vs. 18.86%, *p* = 0.029) [108,111] and associated with higher preoperative calcitonin levels (*p* < 0.001) and more frequent lymph node metastases (*p* < 0.05) compared to patients without *RET* G691S [117]; however, these data were not confirmed in other studies [112,118,119,120].

Finally, Lantieri et al. conducted a meta-analysis including 968 cases (among which 849 were sporadic MTCs) and 2115 healthy individuals. The allelic frequency of *RET* G691S polymorphism was 19.6% in the control subjects and 23.5% among MTC cases, and patients with *RET* G691S polymorphisms exhibited an increased risk of MTC compared to controls (OR = 1.22, 95%CI [1.06–1.39], *p* = 0.0049) [112].

- *RET* L769L (rs1800861)

Several studies speculated that the synonymous *RET* L769L variant may be associated with an earlier age of MTC onset in MEN2 [121]. While some studies have suggested a putative role of the *RET* L769L variant with a higher risk of MTC and younger age of onset [121,122,123,124] as well as a higher risk of lymph node invasion [125], in contrast, a large case-control association study carried out on 585 sporadic MTCs, 1529 non-medullary thyroid cancers, and 989 healthy controls, Gemignani et al. reported that *RET* L769L allele frequency was lower in patients with MTC (OR = 0.70 95%CI [0.58–0.84], *p* = 1.9 × 10^−^^4^) and rare homozygotes showed a lower OR = 0.32 (95%CI [0.17–0.60], *p* = 2.3 × 10^−^^4^) [126]. Finally, Zhang et al. conducted a meta-analysis involving 1117 cases (among which a majority were sporadic MTCs) and 1916 controls from 13 studies and showed a similar frequency of L769L polymorphism in MTC patients and controls, suggesting no association between this variant and an elevated risk of MTC (OR = 1.06, 95%CI [0.94–1.19]) [127].

- *RET* S836S (rs1800862)

Similarly, *RET* S836S polymorphism was found to be more frequent in MEN2A and sporadic MTC patients compared to controls and suspected to be associated with earlier onset and earlier lymph node and distant metastases [128,129,130]. These data were not confirmed by the meta-analysis of Zhang et al., which again found similar frequencies of S836S polymorphism in MTC patients and controls, suggesting no association between this variant and an elevated MTC risk (OR = 1.20, 95%CI [0.97–1.49]) [127].

- Multiple *RET* polymorphisms

Interestingly, *RET* polymorphisms may have an additive effect [131]. Indeed, the *RET* L769L and S836S SNPs were associated with an increased risk of MTC, and individuals harbouring more than three polymorphic alleles exhibited a higher risk of MTC and worse MTC outcomes, suggesting an additive effect of these variants. Additionally, the latter had an increased risk of lymph node and distant metastases at diagnosis [131]. However, another study highlighted contrasting data and showed that in carriers and non-carriers of the *RET* variants (G691S, L767L, S836S, and S904S), sporadic MTCs were clinically and histologically indistinguishable [120].

- Other *RET* polymorphisms

Apart from these variants in the coding sequence, some intronic polymorphisms have been suspected to be associated with a high risk of sporadic MTCs and more aggressive behaviour: IVS1-126G>T (intron 1, c.74-126G>T, rs2565206) [118,132], IVS1-126, and IVS14-24 (intron 14, c.2608-24G>A, rs2472737) [113]. In G533C MEN2A patients, Tamahana et al. showed that IVS14-126G>T was associated with a younger age at diagnosis and IVS8+82A>G (intron 8, c.1648+84G>A, rs3026750); 85–86 insC (intron 6, c.1648+88delC, rs754105711) was associated with the presence of lymph node metastases at diagnosis [133]. In silico analysis predicted that the IVS8+82A>G; 85–86 insC could alter splicing and create a site for NFAT transcription factor (nuclear factor activated T-cells), belonging to a protein family that has been found to be involved in cell cycle regulation, differentiation, survival, and invasion [133]. However, since no in vivo or in vitro experiments are so far available, these data remain inconclusive.

Cebrian and colleagues reported that STOP+388bp G>A (c.*388G>A, rs3026782) was associated with an increased risk of MTC while non intronic *RET* A45A (c.135A>G, p.Ala45=, rs1800858, gnomAD allelic frequency 73.6%) was associated with a protective effect [111], but these results were not replicated in another study [134].

Taken together, these data highlight that *RET* polymorphisms are potentially involved in MTC development, but to date only association studies are available and data concerning putative mechanisms remain elusive [135].

b. Multiple mutations

Yang and colleagues reported that the likely benign *RET* V292M variant may modify the natural history of MTC in the *RET* C634Y family. All three carriers of the *RET* V292M variant were asymptomatic and did not display any manifestation of MTC despite being aged 58, 62, and 82 years. Among *RET* C634Y mutation carriers, patients with the *RET* V292M variant exhibited more extensive disease including lymph node invasion. However, *RET* C634Y belongs to the high-risk grade variants and is thus sufficient to explain this phenotype alone. In vitro studies showed that the *RET* V292M/C634Y double mutant had a faster rate of migration than either single *RET* C634Y mutant. Thus, the authors suggested that the *RET* V292M variant could modify the natural history of MEN2A-related MTC [136]. This mutation has been previously reported in a whole exome study performed by Qi et al. In this study, the authors showed that the combined *RET* mutation p.C634Y/V292M/R67H/R982C exhibited a more aggressive clinical phenotype than p.C364Y or p.V292M/R67H/R982C [136]. The *RET* variant V292M has already been reported in a 44-year-old man with unilateral MTC and unilateral pheochromocytoma, suggesting MEN2 syndrome [137]. However, the allele frequency of V292M is very common (gnomAD frequency 0.06% and up to 0.7% in East Asia) for an uncommon disease. Taken together, the *RET* V292M variant is still therefore considered as a variant of unknown significance (VUS) [40].

c. Somatic mutation in MEN2-associated MTC

- *RET* somatic alterations in MEN2-associated MTCs

While germline and sporadic *RET* mutations lead to MEN2 and sporadic MTC, respectively, both germline *RET* variants and additional somatic *RET* mutation, especially *RET* M918T, in the same patient have been only rarely reported. The impact of this mutation on the clinical course is unknown, and, in practice, a somatic event is rarely looked for in MEN2A patients. Marsh et al. reported, in 1996, cases of MEN2A patients harbouring a germline heterozygous *RET* variant and a second *RET* M918T mutation at the somatic level. Among the thyroid specimens from 16 MEN2A patients (15 MTCs and 1 HCC without MTC), they found four patients with a somatic *RET* M918T mutation: two had germline high-risk variants, C634R and C634S, and the other two had germline moderate-risk variants, C618R and 620R. Three of the four patients, the exception being a young patient with CCH at 11 years old, who was germline-tested through family genetic testing, had metastatic disease. In the 12 thyroid specimens without a somatic *RET* M918T mutation, no other somatic variants were found [138]. In the same year, Eng et al. reported a somatic *RET* M918T mutation in a MEN2 patient with a *RET* C634R germline mutation and with metastatic disease [139]. In these cases, however, the involvement of the M918T somatic variant in the metastatic phenotype could not be confirmed as patients with C634 variants frequently present with aggressive MTC [4]. Lombardo et al. described a 12-year-old girl exhibiting an extensive and invasive MTC despite carrying a germline moderate-risk 804 *RET* variant, leading to the identification of a somatic *RET* M918T mutation [140]. Our team also reported the case of a 35-year-old woman presenting with a particularly aggressive MTC with *RET* L790F germline mutation and in which a *RET* M918T mutation was found at the somatic level [141]. In 2001, Quadro et al. reported a somatic deletion of *RET* exons 4 to 16 in an 18-year-old woman harbouring a locally advanced MTC in which germline *RET* C634R was identified [142]. She died at the age of 27 years due to the progression of lung metastases. This event was located on the WT allele and was limited to the metastatic tumours. Even though the molecular effect of this structural *RET* alteration is unknown (loss or gain of function), it may explain the dramatic outcome in this patient.

Aside from somatic mutations, other somatic alterations may contribute to MTC tumourigenesis in MEN2. As *RET* acts as an oncogenic driver, *RET* copy number alterations have also been assessed in MEN2-related MTC. Koch et al. reported an allelic imbalance of the mutated and wild-type *RET* alleles in 6 of 19 samples and suggested that allelic imbalance could act as a “second activating hit” in *RET* mutant tumours [66]. Similar results were reported later [143,144]. Recently, Bim et al. reported somatic *RET* CNA in the form of retroposed copies in MTC samples and cells lines, suggesting that the generation of somatic *RET* retrocopies may have implications for MTC tumourigenesis and progression [145].

- Non-*RET* mutations could modify MEN2-related MTC outcome

Recently, Mathiesen et al. reported the case of a young woman with the *RET* L790F variant presenting with MTC and lymph node metastases at 14 years of age. She was subsequently treated with surgery and cytotoxic chemotherapy. After a further increase in calcitonin more than ten years after the initial surgery, she presented with histologically-confirmed metastases in mediastinal lymph nodes and probable metastatic lung, mammary, liver, and abdominal lesions. No *RET* somatic mutation was identified, but a variant of *FTL3* was identified. As *FTL3* encodes a transcription factor involved in growth and differentiation, the authors suggested that this variant could have modified the natural history of MTC in this patient [146].

d. Epigenetic alterations in MTC

Epigenetic control of gene expression plays a major role in tumourigenesis and progression in many cancers. In cells, gene expression is under the control of epigenetic regulation that includes DNA methylation, histone modifications, and expression of miRNAs. Epigenetic disturbances can deregulate gene expression and lead to tumourigenesis. The epigenetic landscape in MTCs remains poorly understood [147]. In colorectal cancer, patients with *RET*-hypermethylated tumours showed significantly worse overall survival [148]. On the opposite, *RET* hypomethylation was found in *RET*-WT MTCs compared to normal tissue [149]. Global methylation levels were reported to be higher in MTCs as compared to PTCs, but were not associated with clinical and/or oncological characteristics of the disease [150]. The expression of EZH2 and SMYD3 histone methyltransferase was found to be increased in an aggressive MTC [151]. Several studies have also shown dysregulation of miRNAs in MTC, including an increase in hsa-miR-375 expression. The hsa-miR-375 microRNA is suspected to be anti-proliferative and, in some cases, to have a pro-apoptotic action in some cancers. In vitro, hsa-miR-375 increased the sensitivity of Nthy-ori 3-1 cells to Vandetanib, a tyrosine kinase inhibitor (see below), and may be considered a biomarker of sensitivity [152].

e. Parent-of-origin effects as phenotype modifiers in MEN2-related MTC

There is little information on the impact of parent-of-origin effects on the phenotypic expression of MEN. In the 1990s, Carlson et al. showed that de novo *RET* M918T mutation always occurred on the paternally-derived chromosome, suggesting a differential susceptibility of *RET* to mutation in parental-derived DNA, but showed at the same time that *RET* does not map to a known imprinted locus and revealed that both parental *RET* alleles are expressed in MEN2-related tumours [153]. Recently, in an observational study involving 405 patients carrying heterozygous MEN2A *RET* missense mutation, Machens et al. highlighted that offspring who inherited the trait from the father developed node metastases (*p* = 0.007) at a significantly younger age than offspring who inherited the trait from the mother [154], but the two groups were heterogeneous with regard to the number of C634 high-risk variant carriers. In another study involving 169 MEN2A relatives carrying C634 variants with information available on the gender of the parent who transmitted the familial *RET* variant, the same team reported similar results. Particularly in terms of MTC, they showed that offspring from affected fathers more often showed lymph node metastases (45 vs. 19%, *p* = 0.006) compared to offspring from affected mothers [155]. However, these data should be treated with caution due to the heterogeneity in long-term follow-up between the “paternal-affected” and “maternal-affected” groups [155].

## 6. Targeting *RET* in MTC: A New Therapeutic Paradigm

Historically, advanced progressive MTC had very poor outcomes due to the low response rate to available therapy. Since MTC is derived from C-cells, which do not take up iodine, treatment with radioactive iodine (^131^I) is pointless. Covalent linkage to meta-iodobenzylguanidine could potentially increase the uptake by some MTCs, however this does not offer much in terms of efficacy. Other systemic treatments, such as internal vectorised radiotherapy, using either bispecific antibodies targeting CEA associated with ^131^I or octreotide analogues labelled with yttrium 90, seem only to reduce progression, at the frequent cost of renal and haematological toxicity [156]. Cytotoxic chemotherapy, doxorubicin as a simple drug or in combination with cisplatin, has provided a response rate of between 0 and 20%, though the best response was only partial and median survival was low [156,157]. The association of 5-fluorouracil with dacarbazine seems to be the most effective and the least toxic combination, with a response rate of about 20% [156,157].

Thus, the emergence of tyrosine kinase inhibitors represented a game changer in the management of patients harbouring a progressive advanced MTC, particularly in *RET*-mutant MTCs.

- Multikinase inhibitors

- Vandetanib (ZD6474)

Vandetanib was the first treatment to obtain FDA approval in 2012 for the treatment of symptomatic or progressive MTC in patients with locally advanced or metastatic disease [158]. Vandetanib, a selective oral inhibitor of VEGFR, EGFR, and *RET* [159,160], has been reported to block the enzymatic activity of RET-derived oncoproteins and decrease the proliferation of a *RET*-mutant MTC cell line in vitro [161,162]. It was first assessed in patients with unresectable, locally advanced, or metastatic hereditary MTC, where Vandetanib led to either stable disease or a partial response in around 75% of cases. Further studies demonstrated that Vandetanib resulted in a disease control rate of 68% to 73%, with a safety profile consistent with the initial study. Specifically, treatment-associated adverse effects occurred in about 65% of cases, the most common being diarrhoea, rash, asthenia, and nausea [163,164]. Wells et al., in the phase III ZETA trial, reported that Vandetanib at a dose of 300 mg/d increased progression-free survival (PFS) to 30.5 months, compared to 19.3 months in the placebo arm, in patients with locally advanced or metastatic MTC [165]. Interestingly, a superior treatment-related response was observed in *RET*-positive MTCs, both hereditary and somatic. Somatic level *RET* M918T mutant MTCs exhibited a higher response rate [165]. Real-world data has since shown results consistent with the phase III trial, both in terms of efficacy and safety [166,167,168].

- Cabozantinib (XL184)

Cabozantinib is a multikinase inhibitor targeting MET, VEGFR2, FLT3, c-KIT, and RET, which has exhibited robust antiangiogenic, antitumour, and anti-invasive effects in preclinical models [169] and clinical activity in patients with solid carcinomas. Early observations suggested that Cabozantinib was effective in MTCs and had an acceptable safety profile [170]. Further studies demonstrated that Cabozantinib at a daily dose of 140 mg increased both OS and PFS [171,172,173]. In the phase III EXAM trial, which evaluated locally or distant progressive MTC, Cabozantinib at a daily dose of 140 mg increased PFS by 11.2 months compared to 4.0 months in the placebo group. The 1-year PFS rate slightly increased from 7.2% on placebo to 47.3% with treatment [171]. In a phase III trial, tumour responses confirmed that Cabozantinib is clinically active in all MTC subgroups, regardless of the genotype, with ORRs ranging from 34% (*RET* M918T positive subgroup) to 20% (*RET* M918T negative subgroup), while no objective responses were noticed in patients who received the placebo [172,173]. Cabozantinib-associated adverse events included diarrhoea, palmar–plantar erythrodysesthesia, weight loss, anorexia, nausea, and fatigue which led to dose reduction and treatment discontinuation in 79% and 16% cases, respectively [171]. Though Cabozantinib appears to outperform Vandetanib, the results of phase III studies are not directly comparable since the ZETA trial also included patients without progression and permitted cross-over if progression occurred in the placebo arm, both of which were not permitted in the EXAM trial. Moreover, about 20% of patients in the EXAM trial were previously treated with TKI, including the half who were treated with Vandetanib.

In order to decrease treatment toxicity and related side effects leading to treatment discontinuation, a recent phase IV randomized non-inferiority trial was performed. Patients with progressive metastatic MTC were randomized to Cabozantib at 60 mg/day or 140 mg/d. Severe adverse events, dose reduction, and treatment discontinuation due to side effects were lower for the 60 mg/d group compared to 140 mg/d (63% vs. 72%; 69% vs. 81% and 23% vs. 36%). However, PFS non-inferiority of Cabozantinib at 60 mg/d vs. 140 mg/d was not met (median PFS 11.0 vs. 13.9 months, HR 1.24, *p* = 0.19) [174]. Koehler et al. retrospectively reported real-practice clinical outcomes from 48 patients with advanced MTCs who were treated with TKI. Used as a first-line treatment (n = 7), Cabozantinib led to stable disease in 43% of cases, with a PFS of 9 months. The 6- and 12-months survival rates were 100% and 100%, respectively. As a second-line treatment (n = 16), Cabozantinib achieved a partial response in 31%, with a PFS of 4 months and an OS of 19 months, with 6- and 12-month survival rates of 77% and 70%, respectively. Analysing first- and second-line treatments together, the median PFS was 4 months, and the 6- and 12- months OR rates were 78% and 70%, respectively. These data suggests that Cabozantinib performed less well than Vandetanib. However, only one patient received the recommended dose of 140 mg/d of Cabozantinib compared to over half the patients for Vandetanib [168].

b. RET specific inhibitor

- Pralsetinib (BLU-667)

Pralsetinib is a selective *RET* inhibitor approved in 2020 for the treatment of various *RET*-associated cancers, targeting both wild-type and kinase-activating *RET* mutations [175,176]. It has shown a potent and durable antitumour activity in RET-mutated MTCs in the ARROW trial. In this early phase I/II multicentric open-labelled study involving 122 patients with advanced *RET*-mutant MTCs, Pralsetinib at a dose of 400 mg/day led to an overall response rate of 60% in 55 previously treated patients and 71% in 21 treatment-naïve patients. Disease control rates were 93% and 100%, respectively. Response rates were consistent, with a response rate at 12 months of 92% and 84%, in the two patient groups, respectively. Common adverse effects, which included anaemia, neutropenia, musculoskeletal pain, constipation, increased aspartate amino-transferase, and hypertension, were often mild and led to treatment discontinuation in only 4% of cases. Unfortunately, one patient died, secondary to interstitial pneumonitis, and this was recognized as a treatment-related adverse event [177].

- Selpercatinib (LOXO-292)

Selpercatinib received FDA approval in 2020 for *RET*-associated cancers [178] and may well become a cornerstone in the management of advanced *RET*-positive MTC. Selpercatinib, a highly selective *RET*-specific inhibitor, demonstrated potent activity in preclinical studies against human cancer cell lines harbouring endogenous *RET* gene alterations [179]. This compound was then evaluated in RET-associated human cancers in the LIBRETTO-001 trial. In this phase I/II trial, 143 participants harbouring a *RET*-mutant MTC (55 previously treated with a multikinase inhibitor) received a dose of 20 to 240 mg twice daily (phase 1) or 160 mg twice daily (phase 2). In treatment-naïve patients (n = 88), the objective response rate was 73% and the 1-year sustained objective response rate was 92%, slightly better than 69% and 82%, respectively, that was found in previously treated patients (n = 55). The most common grade 3 or higher adverse events were hypertension, liver enzyme increase, hyponatremia and diarrhoea. The drug-related discontinuation of the treatment remained low (2%) [180]. LIBRETTO-0531, a head-to-head phase III comparison study, is currently underway and will assess both the efficacy and the safety of Selpercatinib at a dose of 160 mg twice daily, compared to Cabozantinib (140 mg/d) or Vandetanib (300 mg/d) [181].

- Zeteletinib (BOS172738)

The most recent compound, Zeteletinib, formerly BOS172738, is a next-generation inhibitor with nanomolar potency against *RET* and far higher selectivity against VEGF2. This molecule is currently in a phase 1 dose-escalation trial involving patients with advanced solid tumours with *RET* gene alteration, including MTC. The first results have shown that BOS172738 had a favourable safety profile in 67 patients with long-term administration, with the most frequent adverse effects classified as mild. The overall response rate was 44% in MTC (n = 16), including one complete response [182]. This trial is still underway and results should be available in the course of 2024 [183].

c. TKI limitations

While these therapies have shown very promising results, recent case reports have suggested a risk of acquired resistance to these drugs, mainly driven by acquired somatic *RET* variants. Bruce et al. reported the case of a patient with metastatic sporadic MTC with a somatic *RET* M918T mutation, who was treated using several therapeutic lines. After Vandetanib and Cabozantinib, he was given Selpercatinib for 12 months and showed a partial response. After 12 months, progression occurred and he was then enrolled in a phase I/II Pralsetinib trial, but after 6 weeks showed rapid deterioration with worsening liver metastases observed on imaging, consistent with progressive disease. Genetic screening performed on a tumour biopsy specimen after Selpercatinib but before Pralsetinib revealed *RET* M918T, *RET* V804M, *RET* G810S mutations, and lastly a *CDKN2A* LOH in addition to the original *RET* mutation [184]. Subbiah et al. published a case report of a 49-year-old man presenting with metastatic sporadic *RET* M918T MTC, treated with several TKI lines, who developed *RET* variants leading to resistance. After consecutive therapy with Sorafenib, Vandetanib, Cabozantinib, and RXDX-105, the authors identified a *RET* V804M variant. The patient was treated with Selpercatinib with PR over 24 months before progression occurred. Genetic analysis performed on blood tumoural DNA identified a *RET* Y806C variant at 2 months, which was missing on subsequent analyses up to 29 months, at which time it was again present. At 22 and 24 months, *RET* G810C and G810S variants were identified, respectively [185].

**Figure 6:**
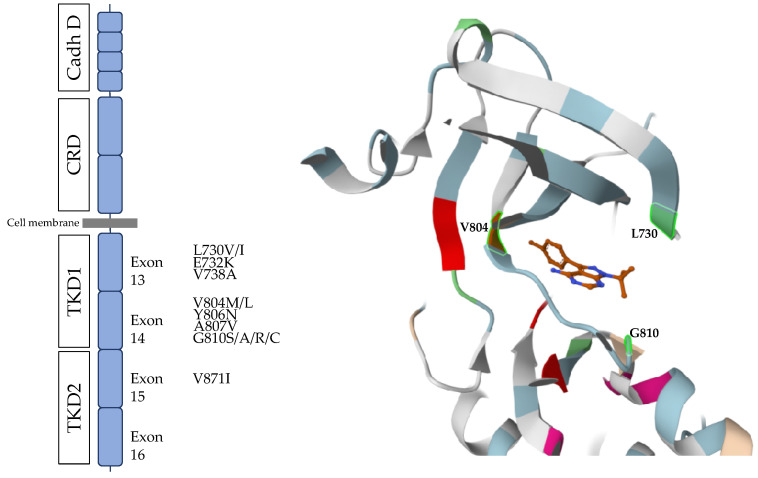
*RET* mutations responsible for tyrosine kinase resistance. CRD: cysteine-rich domain; TKD1: tyrosine kinase domain 1; TKD2: tyrosine kinase domain 2. The left panel represents the different mutations across the *RET* protooncogene. The right panel is a 3D representation of the catalytic site of RET, with ligand (1-(tert-Butyl)-3-(p-tolyl)-1H-pyrazolo [3,4-d]pyrimidin-4-amine) located in the active site. The three residues highlighted in green are the residues V804 (gatekeeper-residue), L730 (rooftop of the pocket), and G810 (solvent front residue). The 3D representation was made by using the Miztli application (https://miztli.biokerden.eu/ (accessed on 23 August 2023).

Most mutations involved in TKI resistance are located at the *RET* catalytic site. The structure of this site is as follows: the N-lobe is composed of beta sheets and an important alpha C helix, which forms the roof and is important for positioning and orientation of the ATP molecule in the catalytic site. An important motif on the N-lobe is the so-called glycine-rich loop that is involved in accommodating the nucleotide in the active site. The region connecting the N- and C-lobes is the hinge. Beyond its structural function, the hinge is involved in ATP binding through hydrogen bonds. This motif is not only important for binding and positioning of the nucleotide but also for the binding of tyrosine kinase inhibitors (TKIs) that compete with ATP. This residue, known as the gatekeeper residue, can restrict nucleotide access to the active site, which in the case of *RET* substitution by a bulkier residue (such as methionine or in some cases leucine) leads to steric hindrance and is associated with resistance to several TKIs, while retaining nucleotide accessibility [186]. The *RET* V804M variant has been reported in early studies as being potentially involved in tyrosine kinase inhibitor resistance. Vitagliano et al. reported that the *RET* V804M variant partially rescued MTC cell proliferation and the MAPK pathway in a MTC cell line harbouring *RET* C634W or M918T mutations treated with Vandetanib [162]. In vitro studies showed that both Cabozantinib and Vandetanib activities are impaired due to the *RET* V804M/L variant [187]. While *RET*-specific inhibitors do not seem to be significantly affected by the *RET* V804 variant in vitro, the *RET* G810 variant appears to markedly reduce Selpercatinib sensitivity and impair Pralsetinib action in *RET* M918T-positive MTC cell lines [185]. Being located at the solvent front, the G810 residue plays a crucial role in nucleotide binding, and replacement of glycine with a larger residue (such as serine, alanine, arginine, or cysteine) leads to steric hindrance and TKI resistance. Apart from the *RET* G810 variant, several other variants in the catalytic site appear to decrease *RET* inhibitor efficacy in vivo and in vitro, including *RET* L730, *RET* V738, *RET* Y806, and lastly *RET* A807 [185,187,188]. In vitro studies have shown that this kinase domain variant impaired the molecule interaction with the catalytic site and decreased *RET* non-specific and specific activity. *RET* variants leading to TKI resistance are outlined in Figure 6.

Other *RET* alterations, such as indels, have been reported as potential modifiers of the treatment response. In the large cohort presented by Elisei et al., two patients harbouring *RET* indels exhibited an excellent response to Selpercatinib [58]. On the other hand, another study reported contrasting data. Wijewardene et al. reported a 35-year-old man presenting with an aggressive metastatic MTC harbouring p.632_633del *RET* that was poorly responsive to Selpercatinib. Structural modelling revealed a paucity of disulphide bonds between C630 and C634 in p.632_633del *RET* sequences, while an intermolecular disulphide bond was formed between two C656 residues. In vitro experiments confirmed a reduction in the efficacy of Selpercatinib upon p.632_633del *RET* compared with wild-type *RET* control [65]. Better understanding and characterisation of *RET* variants leading to TKI resistance is important in order to provide patients with better personalized care, as shown in this report in which liquid biopsy and tissue NGS analyses allowed the author to identify six different *RET* mutations and one *KRAS* mutations, leading to modification of the treatment with another TKI and achieving disease control [189]. Possible factors predisposing to TKI resistance in both sporadic and hereditary MTCs have recently been reported in a study of a cohort of 54 metastatic MTCs with a median follow-up of 10.5 years. Interestingly, a more favourable disease course was found in patients who exhibited serious adverse events under treatment [190].

d. Future perspectives and alternative therapies in MTC

Other drugs and systemic therapies which have shown promising preclinical efficacy are currently being evaluated in phase I/II trials. Among them, new-generation *RET* inhibitors, such as TPX-0046 and HA121-28, and multikinase drug repositioning with Regorafenib, Sorafenib, or even Sunitinib are being evaluated. As MTCs express the somatostatin receptor (SSTR), vectorized internal radiotherapy using 177Lu-SSTR analogue may represent a putative therapy in progressive metastatic tumour. Immune co-inhibitory molecules, such as programmed cell death protein-1 (PD-1/PD-L1) and cytotoxic T-lymphocyte antigen 4 (CTLA-4), are also expressed in MTC tumour cells, thus immune checkpoint inhibitors could also be a possible therapeutic option in progressive MTC. The small molecule ONC201 (imipridone), which inhibits both AKT and ERK, causes apoptotic cell death, decreased transcription of RET, VEGFR2, and IGFBP2, and increased transcription of ATF4 and ATF4 target genes. Since advanced MTCs show low ATF4 expression and ATF4 negatively regulates RET, this drug could be an alternative in *RET* inhibitor-resistant progressive MTC. These compounds and the results of the main studies on their use are summarized by Saltiki et al. [191].

## 7. Conclusions

Medullary thyroid carcinoma is a rare endocrine neoplasm, which occurs in both hereditary (25%) and sporadic (75%) forms. The *RET* proto-oncogene plays a central role in both forms. In hereditary MTC, a germline *RET* mutation leads to MTC development and determines its behaviour. Several putative factors have been suspected to modify the outcome of hereditary MTC, such as *RET* polymorphisms or *RET* somatic mutations. In sporadic MTC, *RET* acts as a tumourigenesis driver in roughly 50% of cases, mainly due to the *RET* M918T somatic mutation. This mutation correlates with MTC aggressiveness and the disease outcome. Regarding MTC therapy, targeting *RET* with non-specific or specific tyrosine kinase inhibitors has produced promising results, leading to FDA approval for four molecules. Follow-up studies will allow us to determine whether new somatic mutations and alternative pathway activation might decrease the efficacy of these drugs on a long-term basis. Aside from *RET*, other molecular markers such as *RAS*, *CDKN*, *MET,* or *ATF4* could also play a key role in both MTC tumourigenesis and therapies. Further studies are now needed in order to improve our understanding of these second line mechanisms.

## Figures and Tables

**Table 1:** *RET* somatic indels reported in sporadic MTCs and associated clinical features.

Ref	*RET* Mutation	Clinical Features
[28]	6-bp del involving codon 630	No clinical data
[50]	6-bp del involving codon 632 to 634	94 yo man, T3N0M0 MTC (post mortem data)
[36]	6-bp del in exon 11 (codons 630–631)	No clinical data
[34]	6-bp del in exon 11 (codons 630–631)	No clinical data
[59]	6-bp del in exon 11 (codons 632–633)	22 yo man, TxN1 MTC,
[60]	9-bp del in exon 11 (codons 633 to 635)	No clinical data
[61]	9-bp del in exon 11 (codons 633 to 635)	58 yo man, no MTC data
[62]	48-bp del in exon 10 (codons 592 to 607)	28 yo woman, T3N1M0 MTC, node progression
[62]	6-bp del in exon 11 (codons 632–633)	32 yo, T4N1b MTC, metastatic progression
[63]	24-bp deletion including codon 634 combined with a 6-bp insertion	No clinical data
[64]	27-bp somatic heterozygous deletion (codons 611–619 or 612–620) in exon 10	46 yo woman, T1NxMx MTC
[51]	6-bp del in exon 11 (codons 632–633)	61 yo woman, T4N1Mx MTC, metastatic progression at 6 months 43 yo man, T4N1Mx MTC, node progression at 12 months 59 yo woman, T4N1Mx MTC, node progression at 36 months
[54]	T636_V637delinsCRT E898_E901del L629_D631delinsH E632_C634del	No clinical data
[58]	c.2694_2705delTGTTTATGAAGA c.2647_2648delGCinsT c.1894_1899delGAGCTG c.1899_1900delGTinsTG	31 patients. MTC showed a rather aggressive clinical behaviour at the time of diagnosis and more frequent metastatic disease during the follow-up. 2 MTC exhibited an excellent response to TKI
[65]	6-bp del in exon 11 (codons 632–633)	35 yo man, aggressive metastatic MTC, poor response to TKI

TKI: tyrosine kinase inhibitor.

**Table 2:** *RET* single nucleotide polymorphisms suspected to be involved in MTC.

# dbSNP	Genomic Coordinates (GRCh37)	HGVS Nomenclature (NM_020975.4)	Total Allelic Frequency (gnomAD v2.1, Last Accessed 4 July 2023)
rs2565206	Chr10:43595781	Intron 1 c.74-126G>T	32.5%
rs1800858	Chr10:43595968	exon 2 c.135A>G, p.Ala45=	73.6%
rs3026782	Chr10:43624105	3′ UTR c. * 388G>A	18.4%
rs754105711	Chr10:43607759	Intron 8 c.1648+88delC *	74.9%
rs3026750	Chr10:43607756	Intron 8 c.1648+84G>A	74.0%
rs1799939	Chr10:43610119	exon 11, c.2071G>A, p.Gly691Ser	20.5%
rs1800861	Chr10:43613843	exon 13, c.2307G>T, p.Leu769=	74.4%
rs1800862	Chr10:43615094	exon 14, c.2508C>T, pSer836=	4.4%
rs2472737	Chr10:43615505	Intron 14 c.2608-24G>A	20.4%
rs1800863	Chr10:43615633	exon 15, c.2712C>G, p.Ser904=	20.6%

* Referred to as IVS8 85_86insC rs3482797 in the literature.

## Data Availability

No new data were created or analysed in this study. Data sharing is not applicable to this article.
